# Identification of the Cell-Surface Protease ADAM9 as an Entry Factor for Encephalomyocarditis Virus

**DOI:** 10.1128/mBio.01780-19

**Published:** 2019-08-13

**Authors:** Jim Baggen, Hendrik Jan Thibaut, Daniel L. Hurdiss, Maryam Wahedi, Caleb D. Marceau, Arno L. W. van Vliet, Jan E. Carette, Frank J. M. van Kuppeveld

**Affiliations:** a Virology Division, Department of Infectious Diseases and Immunology, Faculty of Veterinary Medicine, Utrecht University, Utrecht, The Netherlands; b Department of Microbiology and Immunology, Stanford University School of Medicine, Stanford, California, USA; Columbia University College of Physicians & Surgeons; University of Leeds; University of Wisconsin-Madison

**Keywords:** disintegrin and metalloproteinase domain-containing protein 9 (ADAM9), encephalomyocarditis virus, haploid genetic screen

## Abstract

Encephalomyocarditis virus (EMCV) is an animal pathogen and an important model organism, whose receptor requirements are poorly understood. Here, we employed a genome-wide haploid genetic screen to identify novel EMCV host factors. In addition to the previously described picornavirus receptors sialic acid and glycosaminoglycans, this screen unveiled important new host factors for EMCV. These factors include components of the fibroblast growth factor (FGF) signaling pathway, such as the potential receptors FGFR1 and ADAM9, a cell-surface metalloproteinase. By employing various knockout cells, we confirmed the importance of the identified host factors for EMCV infection. The largest reduction in infection efficiency was observed in cells lacking ADAM9. Pharmacological inhibition of the metalloproteinase activity of ADAM9 did not affect virus infection. Moreover, reconstitution of inactive ADAM9 in knockout cells restored susceptibility to EMCV, pointing to a proteinase-independent role of ADAM9 in mediating EMCV infection. Using neutralization assays with ADAM9-specific antiserum and soluble receptor proteins, we provided evidence for a role of ADAM9 in EMCV entry. Finally, binding assays showed that ADAM9 facilitates attachment of EMCV to the cell surface. Together, our findings reveal a role for ADAM9 as a novel receptor or cofactor for EMCV.

## Abstract (precis)

EMCV is an animal pathogen that causes acute viral infections, usually myocarditis or encephalitis. It is thought to circulate mainly among rodents, from which it is occasionally transmitted to other animal species, including humans. EMCV causes fatal outbreaks of myocarditis and encephalitis in pig farms and zoos, making it an important veterinary pathogen. Although EMCV has been widely used as a model to study mechanisms of viral disease in mice, little is known about its entry mechanism. Here, we employ a haploid genetic screen for EMCV host factors and identify an essential role for ADAM9 in EMCV entry.

## INTRODUCTION

Encephalomyocarditis virus (EMCV) belongs to the genus *Cardiovirus* and the species *Cardiovirus A* within the family of picornaviruses. This virus was first isolated in Florida in 1945 from a gibbon that suddenly died and that was later diagnosed with intense myocarditis and pulmonary edema (1). Following the discovery of EMCV, the virus has been isolated globally from a wide range of animals, including squirrels, elephants, wild boar, antelope, lions, birds, and several nonhuman primate species (2). EMCV emerged as a pathogen in domestic pigs in Europe in the 1990s and has subsequently caused hundreds of outbreaks in pig farms, particularly in Belgium and Italy (3). It is thought that rodents are the natural reservoir of EMCV and that infection of other animal species results from occasional cross-species transmission via contaminated food, water, or carcasses (4). EMCV is associated with sporadic cases and outbreaks of myocarditis and encephalitis, usually among captive animals living in pig farms, zoos, or primate research centers (5). However, pathogenesis of EMCV appears to be dependent on the strain and differs between host species. EMCV infection of nonhuman primates usually results in death due to heart failure, whereas infection of pigs can cause acute myocarditis or reproductive disorders (2). So far, only a few cases of EMCV infection in humans have been reported; in these cases, patients presented with mild symptoms, such as febrile illness, nausea, and headache (5). Nevertheless, several serological studies reported that exposure to EMCV is common in humans (6), particularly among hunters (7).

EMCV has long been used as a model virus to study the mechanisms of viral suppression of the innate immune system for example (8, 9). Nevertheless, only a few studies have investigated the receptor requirements of this virus. The first study chronicling a functional EMCV receptor reported that infection of primary vascular endothelial cells by the EMC-D strain was inhibited by antibodies targeting vascular cell adhesion molecule 1 (VCAM-1), a protein belonging to the immunoglobulin superfamily (10). Moreover, VCAM-1 overexpression increased the susceptibility of CHO cells to EMCV infection. A second study, however, reported that EMCV can infect cells lacking VCAM-1 expression and showed that attachment of the virus to these cells was mediated by a 70-kDa sialoglycoprotein of unknown identity (11). Yet another study reported that infection of primary human cardiomyocytes by one EMCV strain was dependent on sialic acid (Sia) and heparan sulfate, while a second strain did not require these factors. Thus, although several cell-surface molecules have been implicated as possible EMCV receptors, the exact receptor requirements of EMCV are currently still unclear. In this study, we aimed to unveil possible unknown EMCV host factors via an unbiased genome-wide approach and identified the disintegrin and metalloproteinase domain-containing protein 9 (ADAM9) as a novel factor supporting EMCV entry.

## RESULTS

### A genome-wide haploid screen identifies novel host factors for EMCV.

**FIG 1:**
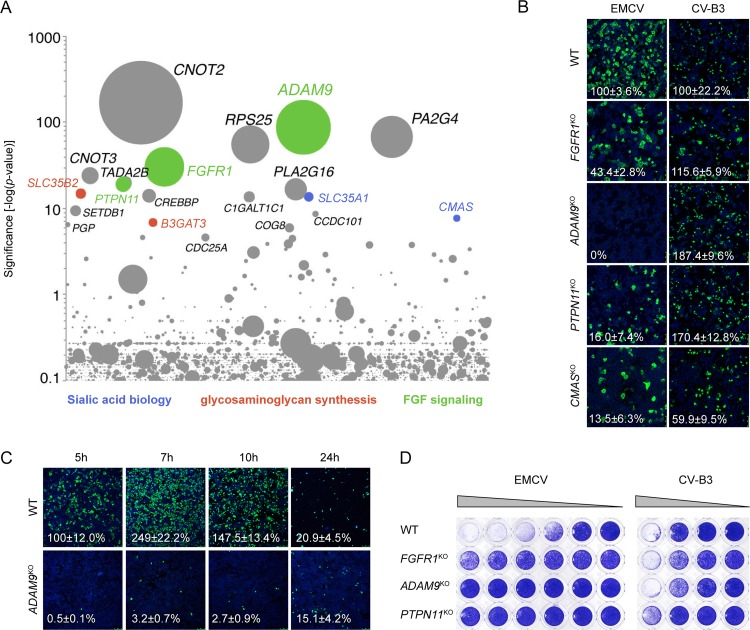
A genome-wide haploid screen identifies novel host factors for EMCV. (A) Bubble plot indicating the significance of enrichment of gene trap insertions in genes (*y* axis) in an EMCV-infected cell population compared to an uninfected control population. Each bubble represents a gene, with size corresponding to the number of gene trap insertions per gene. Genes were randomly distributed on the *x* axis. The names of the top 20 hits are indicated. (B) HAP1 cells deficient for various genes were infected with EMCV or coxsackievirus and adenovirus receptor (CAR)-binding CV-B3, followed by staining of capsid proteins (EMCV) or 3A protein (CV-B3) and nuclei (blue) at 5 h postinfection. Representative confocal micrographs are shown. The values (percentages) in the micrographs are the means ± standard errors of the means (SEM) for ≥3 technical replicates, normalized to the WT value. (C) HAP1 clones were infected with EMCV, followed by staining of capsid proteins (green) and nuclei (blue) at the indicated times postinfection. Representative confocal micrographs are shown. Percentage values denote means ± SEM for two to five technical replicates, normalized to the WT value at 5 h. (D) HAP1 clones were infected with EMCV or CV-B3, followed by crystal violet staining of surviving cells. The experiment was conducted twice with similar results.

Haploid genetic screens have proven to be a powerful method to identify host factors required for virus infection, particularly factors involved in virus entry (12–14). We performed such a screen with EMCV (strain Mengovirus, ATCC VR-1598) and identified, among others, genes involved in sialic acid biology (*SLC35A1* and *CMAS*) and synthesis of sulfated glycosaminoglycans (*B3GAT3* and *SLC35B2*), two glycans that have previously been found to facilitate EMCV binding to cells (15) (Fig. 1A). Among the top hits was also *PLA2G16*, which encodes a phospholipase that was recently identified as a host factor for enteroviruses and cardioviruses and is required for delivery of the viral genome from the endosome into the cytoplasm (14, 16), further confirming the validity of the screen. Other hits include three genes involved in FGF signaling (*ADAM9*, *FGFR1*, and *PTPN11*), two of which encode plausible receptor candidates (ADAM9 and FGFR1). ADAM9, a member of the ADAM (a disintegrin and metalloproteinase) family, is a membrane-anchored protease that promotes tumor progression in a large variety of cancers (17–19) and is known to cleave several membrane proteins, including epidermal growth factor (EGF), fibroblast growth factor (FGF) receptor 2IIIb (FGFR2IIIb) (20), and VCAM-1 (21). Proteolytic cleavage of these proteins by ADAM9 results in shedding of their ectodomains, regulating their signaling activity (22). Fibroblast growth factor receptor 1 (FGFR1) is one of the four mammalian FGF receptors, which regulate many cellular functions by transmitting signals conveyed by extracellular FGFs into the cell via their intracellular tyrosine kinase domains (23). SH2 domain-containing tyrosine phosphatase 2 (Shp2; encoded by *PTPN11*) has been reported to regulate the activity of various receptors, including FGF receptors, by dephosphorylation of their intracellular kinase domains (24). Other screen hits include *CNOT2* and *CNOT3* (transcription regulation), *RPS25* (ribosomal component), and *PA2G4* (growth regulation, possibly translation). Although these genes may be important for EMCV infection, we have focused on potential entry factors in this study.

To confirm the involvement of these factors in EMCV infection, we determined the susceptibility of HAP1 cells lacking expression of *FGFR1*, *ADAM9*, *PTPN11*, or *CMAS* to EMCV. Analysis of the number of infected cells showed that knockout of each gene reduced the susceptibility to EMCV infection, whereas the negative-control virus coxsackievirus B3 (CV-B3) that employs the coxsackievirus and adenovirus receptor (CAR) (Fig. 1B) was hardly affected. The largest reduction in infection efficiency was observed in HAP1 *ADAM9*^KO^ (KO stands for knockout) cells, in which EMCV capsid proteins were not detected at 5 h postinfection. Infection became apparent at later time points, but the infection efficiency was still severely impaired even after 24 h (Fig. 1C). Crystal violet staining of viable cells showed that knockout of *FGFR1*, *ADAM9*, and *PTPN11* protected cells from cytopathic effect induction by EMCV (Fig. 1D). Together, these data reveal that components of the FGF signaling pathway are involved in EMCV infection, including the receptor candidates FGFR1 and ADAM9.

### ADAM9 plays a role in an early step in EMCV infection.

**FIG 2:**
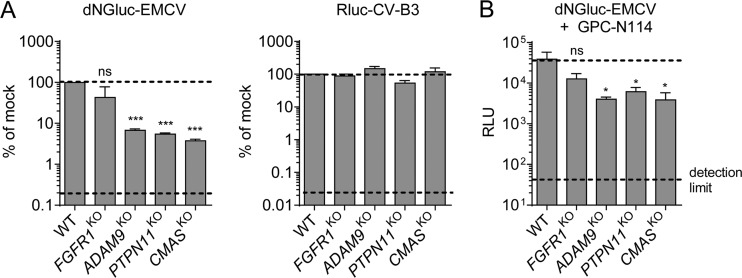
ADAM9 plays a role in an early step in EMCV infection. (A) HAP1 clones were infected with dNGluc-EMCV or Rluc-CV-B3. Luminescence was measured at 6 h postinfection. Dashed lines indicate the WT signal (top) and the signal in the presence of a replication inhibitor (bottom). Values are means plus SEM (error bars) from two independent experiments. Values were compared to the WT values, and statistical significance was calculated by an unpaired two-sided *t* test. Values that are significantly different from the WT value are indicated as follows: ***, *P* < 0.001. Values that are not significantly different (ns) are indicated. (B) HAP1 clones were infected with dNGluc-EMCV in the presence of the replication inhibitor GPC-N114 (10 μM). Luminescence (in relative light units [RLU]) was measured at 6 h postinfection. Dashed lines indicate the WT signal (top) and the signal produced by uninfected cells (bottom). Values are means plus SEM (error bars) for three biological replicates. Statistical significance compared with the WT values were calculated by an unpaired two-sided *t* test of log-transformed data; ns, not significant; *, *P* < 0.05.

To gain insight into the step in the viral life cycle that is facilitated by the identified host factors, we analyzed reporter expression in HAP1 knockout clones by recombinant luciferase expressing EMCV (dNGluc-EMCV) (25) and CV-B3 (RLuc-CV-B3) (26) in the presence or absence of replication inhibitor GPC-N114 (27). Analysis of luciferase reporter expression in the absence of GPC-N114 at 6 h postinfection (i.e., within a single replication cycle) showed that knockout of *ADAM9* and *PTPN11*, but not *FGFR1*, significantly inhibited EMCV infection (Fig. 2A). Similar results were obtained in the presence of GPC-N114, when luciferase is expressed exclusively from incoming viral RNA (Fig. 2B). Knockout of *ADAM9* and *PTPN11* inhibited luciferase expression to a similar extent as *CMAS* knockout, which prevents cell-surface expression of sialic acids and is therefore expected to affect virus entry. Together, these data pointed to a role of ADAM9 in an early step in infection, such as viral entry or genome translation.

### Mouse ADAM9 supports EMCV infection independently of its metalloprotease activity.

**FIG 3:**
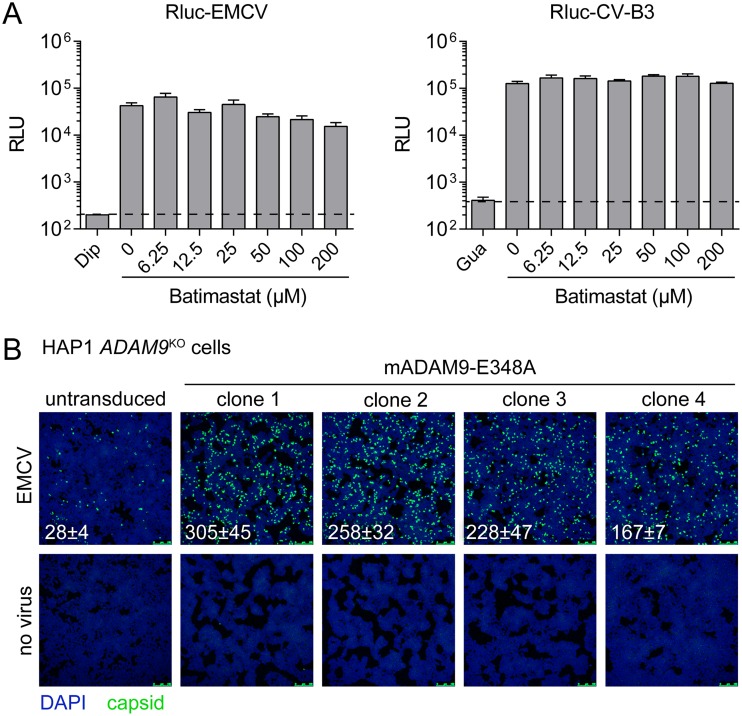
EMCV can use mouse ADAM9 to infect HAP1 cells, and this is independent of its metalloprotease activity. (A) HAP1 clones were pretreated for 30 min with batimastat and infected with Rluc-EMCV or Rluc-CV-B3 in the presence of batimastat. Luminescence was measured at 6 h postinfection. Dashed lines indicate the signal in the presence of the replication inhibitors dipyridamole (Dip) or guanidine hydrochloride (Gua). Values are means plus SEM (error bars) for four biological replicates. (B) HAP1 *ADAM9*^KO^ cells were transduced with murine leukemia virus particles harboring plasmid encoding catalytically inactive ADAM9 mutant E348A. Transduced cells were selected with hygromycin and subcloned. Cells were infected with EMCV, followed by staining of capsid proteins (green) and nuclei (blue). Representative confocal micrographs are shown. The values in the micrographs are mean ± SEM of the number of infected cells per field for two technical replicates.

To investigate whether EMCV infection requires the metalloprotease activity of ADAM9, we used the broad-spectrum metalloprotease inhibitor batimastat, which targets many matrix metalloproteases (MMPs) and ADAM proteins, including ADAM9 (28). Batimastat barely inhibited infection of HAP1 cells by *Renilla* luciferase-expressing EMCV (25) and CV-B3 at concentrations up to 200 μM, which is a concentration 4,000-fold higher than the 50% inhibitory concentration (IC_50_) value reported for ADAM9 inhibition in cell-based assays (29). To further prove that the metalloprotease activity of ADAM9 is dispensable for EMCV infection, we overexpressed mouse ADAM9 carrying the E348A substitution (mADAM9-E348A), a substitution that renders the protein catalytically inactive (20). Transduction of HAP1 ADAM9^KO^ cells with mADAM9-E348A and subsequent infection of several clones with EMCV showed that mADAM9-E348A overexpression increases the susceptibility of human ADAM9-deficient cells to EMCV (Fig. 3). Together, these data indicate that EMCV can use both human and mouse ADAM9 to infect cells and that its metalloprotease activity is dispensable for virus infection.

### ADAM9 is required for the entry phase of EMCV infection.

**FIG 4:**
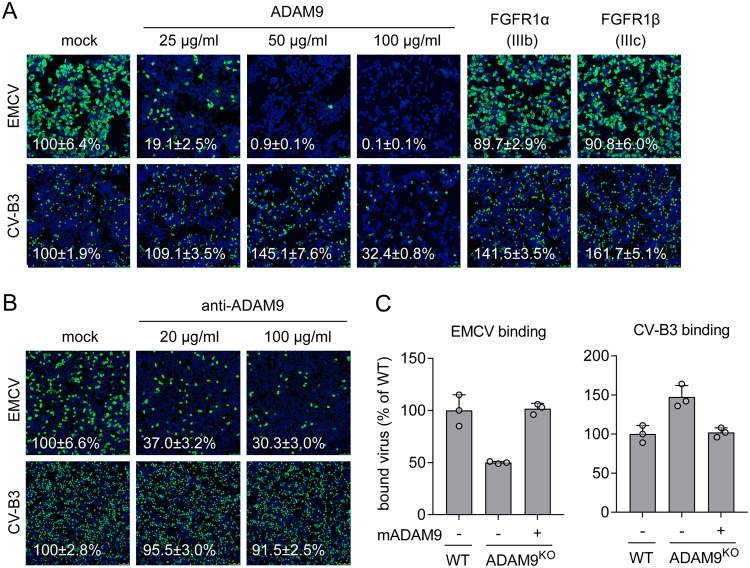
ADAM9 is required for the entry phase of EMCV infection. (A) EMCV or CV-B3 were pretreated with the indicated concentrations of soluble ADAM9 protein or 100 μg/ml soluble FGFR1α/FGFR1β proteins for 1 h at 37°C and used to infect HAP1 cells. Capsid proteins (EMCV) or 3A protein (CV-B3) and nuclei (blue) were stained at 7 h postinfection. (B) HAP1 cells were pretreated with the indicated concentrations of antibody targeting ADAM9 and infected with virus for 7 h, followed by staining as described above for panel A. Representative confocal micrographs are shown in panels A, B, and C. Values (percentages) on the micrographs are mean ± SEM values for 3 or 4 (A) or 4 (B) technical replicates, normalized to the value for mock treatment. (C) WT, ADAM9^KO^, or ADAM9^KO^ HAP1 cells overexpressing mouse ADAM9 mutant E348A (mADAM9) were incubated with EMCV or CV-B3 on ice, followed by qPCR analysis of bound virus. The values are means plus standard deviations (SD) (error bars) for three biological replicates. Experiments were conducted twice with similar results.

Since ADAM9 and FGFR1 are membrane proteins that could potentially serve as EMCV receptors, we tested whether soluble forms of these proteins could neutralize EMCV infection of cells. Preincubation of EMCV with soluble ADAM9 protein (up to 50 μg/ml) inhibited infection of HAP1 cells, without having adverse effects on CV-B3 infection or cell morphology (Fig. 4A). In contrast, virus incubation with two soluble FGFR1 splicing variants (FGFR1α IIIb and FGFR1β IIIc) did not affect EMCV infectivity. These data suggest that soluble ADAM9 either directly binds to the virus or, alternatively, needs to be cointernalized with the virus to exert its inhibitory effect.

Next, to establish whether ADAM9 expressed on the cell surface is required for EMCV infection, we neutralized infection with an ADAM9-specific antibody. Antibody pretreatment inhibited infection of HAP1 cells with EMCV, but not with CV-B3 (Fig. 4B), indicating that the support of EMCV infection by endogenous ADAM9 takes place at the cell surface or in the endocytic compartment. Finally, quantitative PCR (qPCR) analysis of virus attachment showed that ADAM9 knockout inhibits binding of EMCV, but not CV-B3, to HAP1 cells and that binding can be restored by overexpression of mADAM9-E348A (Fig. 4C). This result reveals that the catalytically inactive ADAM9 contributes to the initial attachment of EMCV to the cell surface. Altogether, these data show that ADAM9 is a host factor required for the entry phase of EMCV infection.

## DISCUSSION

In this study, we employed a genome-wide haploid genetic screen to identify host factors for EMCV, which pointed toward a role of sialic acid, glycosaminoglycans, and components of the FGF signaling pathway. Using knockout cell lines, we confirmed the involvement of ADAM9, Shp2, FGFR1, and CMAS in EMCV infection. Via chemical inhibition and overexpression of inactive ADAM9, we showed that ADAM9 supports EMCV infection independently of its metalloprotease activity. Finally, virus neutralization experiments and analysis of virus binding to ADAM9^KO^ cells showed that ADAM9 plays a role in EMCV entry.

Sialic acid and glycosaminoglycans have been described as cell attachment receptors for several picornaviruses, including enterovirus A71 (30, 31), EMCV strain 1086C (15), and the related cardiovirus Theiler’s murine encephalitis virus (32, 33). For viruses that use multiple receptors, these glycans usually serve as secondary receptors (30, 34, 35) that enhance infection by concentrating the virus on the cell surface. Therefore, it is plausible that EMCV uses sialic acid and glycosaminoglycans to enhance attachment, while the virus engages a protein receptor for internalization and subsequent uncoating. Two protein receptor candidates identified in the haploid screen are ADAM9 and FGFR1. Only soluble ADAM9 was able to inhibit EMCV infection, whereas treatment with soluble FGFR1 variants did not affect EMCV infection. However, it should be noted that this lack of an inhibitory effect might be due to the use of stable FGFR1 homodimers, which may not be able to interact with the virus. Our observation that soluble ADAM9 reduced virus infection hints toward a direct interaction. However, in immunoprecipitation assays, biolayer interferometry experiments, and cryo-electron microscopy analysis, we found no evidence for a direct interaction between EMCV and ADAM9 (data not shown). Hence, whether ADAM9 serves as a bona fide receptor facilitating virus internalization and uncoating remains to be established. Our finding that ADAM9 facilitates EMCV attachment to the cell surface may also be interpreted as evidence for a direct interaction, but other scenarios cannot be excluded. For instance, it is possible that ADAM9 function as a co-receptor that complexes with other cell-surface proteins prior to virus binding or that it serves as a cofactor that does not interact with the virus itself. Besides a metalloprotease domain, ADAM9 possesses other domains that could be involved in EMCV entry, including an EGF-like domain, a cysteine-rich region, and a disintegrin domain (36). For instance, the disintegrin domain of ADAM9 has been shown to regulate cell adhesion by interacting with multiple different β1 integrins (37, 38). By sequestering β1 integrins, ADAM9 could promote the accessibility of other β1 integrin-binding proteins on the cell surface, including possible EMCV receptors such as VCAM-1. More research is needed to decipher the exact role of ADAM9 in the entry process of EMCV.

While this article was being prepared, ADAM9 was identified as an essential factor for EMCV entry in another study, which used a genome-wide CRISPR/Cas9-based screen in HeLa cells (39). Knockout of ADAM9 strongly inhibited infection of different EMCV strains in both human and mouse cells, and this inhibition could be bypassed by transfection-mediated delivery of viral RNA to the cytosol, supporting the idea that ADAM9 is required for virus entry. Furthermore, consistent with our finding, it was shown that EMCV infection was rescued with similar efficiency by expression of wild-type (WT) and catalytically inactive ADAM9. These findings, together with the results of our study strongly establish that ADAM9 is required for the early stage of EMCV infection.

The identification of FGFR1 in our haploid screen and the fact that it belongs to the immunoglobulin-like (Ig-like) superfamily, like most picornavirus protein receptors, suggested that FGFR1 could serve as an EMCV receptor. However, the effect of FGFR1 knockout on EMCV infection was relatively small. Although no other receptor candidates were found in the screen, other receptors (e.g., other FGFRs) or other cell-surface proteins may also be involved in EMCV entry. Previously, VCAM-1, an Ig-like sialyglycoprotein, was implicated as an EMCV receptor (10). We did not identify VCAM-1 as a hit in our haploid screen, but this may be explained by the fact that expression of VCAM-1 is limited to specific cell lines and is relatively low in HAP1 cells (40). We also identified the phosphatase Shp2 as a factor involved in EMCV entry. This phosphatase is involved in intracellular signaling by several cellular receptors, including FGFRs, and was previously found to mediate poliovirus infection by association with its receptor (41). The exact role of Shp2 in the internalization of EMCV remains to be established. Although the role of FGFR1 in EMCV entry remains unclear, the possible link between ADAM9 and FGFR1 identified in this study is remarkable and may point to some physiological connection. Both ADAM9 and FGFR1 are important factors in tumor development (29, 42) and metastasis induction (19). ADAM9 was previously shown to cleave FGFR2 IIIb (20), but FGFR1 was never implicated as an ADAM9 substrate. Whether and how ADAM9 regulates FGFR1 activity remains to be established.

In conclusion, ADAM9 plays either a direct role in EMCV entry as a receptor or an indirect role as a cofactor. In the future, identification of cell-surface proteins that directly bind EMCV, for example by chemical cross-linking and mass spectrometry, might shed more light on the functions of the different EMCV host factors identified here. Also, it should be investigated whether EMCV relies on ADAM9 in physiologically relevant cell types, such as primary vascular endothelial cells. Because mice lacking ADAM9 are viable and were not found to have abnormalities (43), ADAM9 could be considered a potential target for antiviral therapy.

## MATERIALS AND METHODS

### Cells, viruses, and reagents.

HAP1 *CMAS*^KO^, *FGFR1*^KO^, *PTPN11*^KO^, *ADAM9*^KO^, and wild-type (WT) HAP1 cells were obtained from Horizon Discovery Group plc (Cambridge, UK) and cultured in Iscove’s modified Dulbecco’s medium (IMDM) (Lonza) containing 10% (vol/vol) fetal calf serum (FCS). HEK293T (ATCC CRL-3216) cells were cultured in Dulbecco’s minimum essential medium (DMEM) (Lonza) supplemented with 10% (vol/vol) FCS. All cells were tested for mycoplasma contamination. The EMCV (strain mengovirus vMwt) used for the haploid genetic screen was obtained from the American Type Culture Collection (ATCC VR-1598), while in all other experiments, the EMCV strain mengovirus vM16.1 was used. CV-B3 (Nancy) and EMCV (vM16.1) were obtained by transfecting *in vitro*-transcribed RNA derived from full-length infectious clones p53CB3/T7 and pM16.1, respectively. Rluc-EMCV and Rluc-CV-B3 consist of viral genomic RNA encoding *Renilla* luciferase upstream of the leader sequence (RLuc-EMCV) or upstream of the P1 coding sequence (Rluc-CV-B3) (25, 26). In dNGluc-EMCV, *Renilla* luciferase was replaced by an N-terminal deletion mutant of *Gaussia* luciferase (dNGluc) to minimize the size of the viral genome. The following chemicals and reagents were used in this study: recombinant human FGFR1α IIIb (catalog no. 655-FR; R&D Systems), recombinant human FGFR1β IIIc (catalog no. 661-FR; R&D Systems), recombinant human ADAM9 (catalog no. 939-AD-020; R&D Systems), goat polyclonal anti-ADAM9 (catalog no. AF939; R&D Systems), and batimastat (catalog no. 2961; Tocris).

### Haploid genetic screen with EMCV.

The haploid genetic screen was performed as described previously (44). Briefly, HAP1 cells were gene trap mutagenized, expanded, and exposed to EMCV. After selection with virus, cells were expanded, and genomic DNA was isolated, followed by sequence analysis. For each gene, the enrichment of insertion sites was calculated by comparing the EMCV-selected population with a noninfected control population.

### Infectivity assays.

Cells were incubated with virus for 1 h at 37°C, supplied with fresh medium, and incubated at 37°C for 6 h (luciferase assays) or 7 h (immunofluorescence). In neutralization assays, viruses or cells were pretreated with ligands for 1 h at 37°C, unless otherwise indicated. Crystal violet staining was performed 2 days postinfection. For immunofluorescence staining, cells were fixed by submersion in a 4% paraformaldehyde solution for 15 min. Fixed cells were stained with 1:1,000 diluted rabbit antiserum against mengovirus capsids (obtained from Ann Palmenberg, University of Wisconsin) or a 1:100 diluted mouse monoclonal antibody against CV-B3 protein 3A. Cells were examined by confocal microscopy (Leica SPE-II), and the number of infected cells was quantified with ImageJ.

### Generation of murine leukemia virus particles for overexpression of ADAM9 E348A.

The gene encoding catalytically inactive mouse ADAM9 mutant E348A was amplified by PCR from plasmids obtained from Carl P. Blobel (Cornell University, New York). The amplicon was inserted into pQXCIH using restriction enzymes NotI and BamHI. This plasmid was cotransfected with packaging plasmids pCAGGS-VSV-G and pMLV-gag-pol into HEK293T cells. Murine leukemia virus (MLV) particles were harvested after 5 days and used to transduce HAP1 *ADAM9*^KO^ cells. Transgene-expressing cells were selected with hygromycin, after which four single-cell clones were expanded and validated by sequencing the *ADAM9* region containing the inactivating mutation.

### Virus binding assays.

Cells were incubated with virus for 1 h on ice and washed three times with ice-cold phosphate-buffered saline (PBS). RNA was isolated from cells using the NucleoSpin RNA isolation kit (catalog no. 740955.250; Macherey-Nagel). cDNA was generated using the TaqMan reverse transcription reagent kit (catalog no. N8080234; Applied Biosystems). Quantitative PCR was performed using the Roche Lightcycler 480 SYBR green I master kit (catalog no. 04 887 352 001; Roche).
